# Cost analysis of hospitalized children suspected of rare genetic diseases

**DOI:** 10.1186/s13023-025-04182-5

**Published:** 2026-01-13

**Authors:** Jean Martial Kouame, Simon LaRue, Camille Varin-Tremblay, Jacques L. Michaud, Anne-Marie Laberge, Jason Robert Guertin

**Affiliations:** 1 Département de médecine sociale et préventive, Faculté de médecine, Université Laval, 1050, Avenue de la Médecine, Local 2431, Québec, G1V 0A6 Canada; 2 Axe de recherche en santé des populations et pratiques optimales en santé, Centre de recherche du CHU de Québec, Québec, Canada; 3 Centre de recherche Azrieli du CHU Sainte-Justine, Montréal, Canada; 4 Département de pédiatrie, Faculté de médecine, Université de Montréal, Montréal, Canada; 5 Service de génétique médicale, Département de Pédiatrie, CHU Sainte- Justine, Montréal, Canada; 6 Département de médecine sociale et préventive, École de Santé Publique de l’Université de Montréal, Montréal, Canada; 7 Centre de recherche en organogénèse expérimentale de l’Université Laval/LOEX, Québec, Canada

**Keywords:** Cost analysis, Canadian clinical practice, Conventional genetic tests, Rare genetic disorders

## Abstract

**Background:**

Conventional genetic tests (CGT) are currently employed in Canada to investigate rare genetic disorders (RGD) but costs related to their use have been scarcely examined. We aimed to estimate the total hospital costs for children suspected of having rare genetic diseases.

**Methods:**

This was a retrospective study based on the patients’ medical records. The analysis adopted the hospital’s perspective and the total hospitalization cost was assessed using an inhospital cost database, which included direct costs (e.g., lab tests, drugs), and indirect costs (e.g., hospital overheads). We provide mean costs with 95% confidence intervals obtained with bootstrap analyses.

**Results:**

Data from a total of 223 children were analyzed. Mean age was 3.0 years and 119 (53.4%) were male. The average length of hospital stay was 42 days. The mean hospitalization cost per patient in the CGT cohort was $170,337 (95% CI. 128,231 − 219,277). The cost for newborns (0–30 days), $219,498 (95% CI. 144,061–312,395), was higher than for other age categories (*p* = 0.0914).

**Conclusion:**

Hospitalization costs for the CGT cohort were substantially greater than hospitalization costs for the general pediatric population. Subgroup analyses revealed that newborns investigated with CGT were the most expensive among all pediatric cases.

## Introduction

Rare genetic disorders (RGD) and congenital malformations indicating a possible genetic etiology affect 1–2% of live births and are the leading cause of hospitalization and death in infants in Canada [1]. Among children admitted to neonatal or pediatric intensive care units and other units of the hospital, those with a RGD have a higher risk of both mortality and prolonged hospital stay [2–5]. Identifying the underlying genetic diagnosis in such cases is especially important because RGD can evolve rapidly at this early stage of life and a precise diagnosis can have critical implications for an infant or child’s health immediately and for his entire life.

Several factors greatly complicate diagnosing rare diseases in the pediatric population, such as the considerable number of disease-associated genes, the absence of pathognomonic features or signs, especially in young children, and the heterogeneous nature of the underlying pathogenic changes [6]. The process is often lengthy, requiring prompt recognition of suggestive features and timely referral to different specialists, and may require multiple attempts [7, 8]. The main options for diagnostic testing of individuals suspected of having a genetic disorder include chromosomal microarray analysis, targeted sequencing of a limited number of genes (gene panel testing or Conventional Genetic Tests [CGT]), or genome-wide sequencing, such as exome sequencing (ES) and genome sequencing (GS) [9–11]. In Canada, diagnostic genome-wide sequencing has become available only recently and is not yet covered by all provincial health care systems.

According to Gonzaludo et al. [12], aggregate total charges for suspected RGD accounted for $14 to $57 billion (11–46%) of the “national bill” for pediatric patients in the U.S. in 2012. Among patients with RGD, mean total costs of hospitalization were $16,000–77,000 higher (*P* < 0.0001) in neonates and $12,000–17,000 higher (*P* < 0.0001) in pediatric patients than in adult patients [13–15]. Unlike in the U.S., Canadian studies evaluating the cost of hospital stay of patients with RGD are lacking. To the best of our knowledge, only one such study, specific to the genetic investigation of autism, exists in the literature and it did not examine all costs incurred during the patients’ hospitalizations [16].

In this context, the key objective of this study was to provide recent and complete information about the cost of hospitalizations for the investigation of RGD in a Canadian setting. Precisely, we aimed to assess the total cost of the hospital stay of all children admitted to the hospital who were investigated with CGT, and treatment for their diseases from a public pediatric tertiary care hospital in Canada.

## Methods

### Study design

This is a retrospective study using data collected from patients admitted to the CHU Sainte-Justine, an academic pediatric tertiary care hospital located in Montreal (Canada), between January 2021 and November 2022. Our cost evaluation was conducted from a public hospital perspective, which excludes physician fees. Physician fees in Quebec are mostly paid by the province’s Ministry of Health and Social Services and not by the hospital and are therefore excluded from this cost study.

### Population

To be included in our study, patients had to answer all of the following inclusion criteria: (1) age < 18 years old at the time of admission at CHU Sainte-Justine; (2) admitted to the neonatal or pediatric intensive care units and other units of the hospital; (3) had CGT during the hospitalization to investigate for a RGD. Patients who do not reside in the province of Quebec were excluded. These patients are not insured by the provincial healthcare system and thus costs would not be covered in a hospital perspective. Eligible cases were identified from the genetic testing registry of the hospital.

### Data description

Patients were followed from the time of their first admission, until discharge or inhospital death, whichever occurred first *(hereby defined as their index hospitalization)*. Hospital data were used to collect information about the clinical information (i.e., death, diagnostic test, DRG), sociodemographic information (i.e., age, sex) as well as hospitalizations and diagnosis information. Costs were extracted from the CPSS (*Coût par Parcours de Soins et Services*) database, an inhospital administrative costing database. The CPSS provided cost data for each patient’s total hospital stay, including stratified direct (i.e., resources directly used in diagnostics and treatments) and indirect costs (i.e., broader hospital resources allocated to patients, such as electricity expenses and other overhead costs).

### Outcomes

The main outcome was the total cost of the index hospitalization (only the first admission, readmissions, if any, could not be assessed within this study), which included all costs incurred by patients during their hospital stay as well as the costs of diagnostic tests performed in external laboratories. Secondary outcomes include patients’ inpatient length of stay and incidence of death.

### Statistical analysis

Descriptive analysis of patients’ demographic characteristics and clinical outcomes were carried out and developed according to age group. The main reasons for admission in units care were categorized based on Diagnosis-related group (DRG) [17]. Unstratified as well as age- and sex-stratified average costs were estimated. Non-parametric bootstrap with 10,000 iterations was performed to estimate associated 95% confidence intervals [18]. Cost outcomes were compared with robust t-test and robust Fisher test utilizing a bootstrap procedure. The significance level was considered at 5% for the various statistical tests. All cost analyses were undertaken in conformity with the Canadian Agency for Drugs and Technologies in Health guidelines (now known as Canada’s Drug Agency) [19]. Costs were adjusted using the all-item consumer price index and were presented in 2023 Canadian dollars (CAD) [20]. All analyses were performed using R 4.4.2 software.

## Results

### Cohort characteristics

The hospital genetic testing registry identified 246 unique patients who had at least one CGT during their hospitalization. Of these patients, nine were excluded after applying the inclusion and exclusion criteria. Patients’ inpatient data were used to estimate the cost of their hospital stay based on the CPSS framework. Following this analysis, 14 additional patients were excluded because CGT cost data were missing. A total of 223 children were therefore included in our final study population. Cohort characteristics are summarised in Table 1. Patients were 3.0 years old on average (standard deviation 5.3 years), and 119 patients were male (53.4%). Based on DRG, the most common reasons for admission in our cohort were Neonates with genetic disorders: 97 (43.5%); Nervous System Disorders: 23 (10.3%); Digestive System Disorders: 12 (5.4%). The most frequent diagnostic tests requested after evaluation by a geneticist were multigene panel and gene panel.

**Table 1:** Patients’ demographics and clinical characteristics

Variable	Overall *n* = 223	0–30 days *n* = 104	31 days – 365 days *n* = 48	Above 1 year old *n* = 71
Male sex (percentage)	119 (53.4)	57 (54.8)	26 (54.2)	36 (50.7)
Age mean (SD)	3.0 (5.3) years	4 (6) days	119 (83) days	9.3 (5.6) years
Hospital unit where patients were tested				
PICU	27 (12.1%)	3 (2.9%)	8 (16.7%)	16 (22.5%)
NICU	64 (28.7%)	61 (58.7%)	3 (6.2%)	0 (0.0%)
NIntCU	27 (12.1%)	23 (22.1%)	4 (8.3%)	0 (0.0%)
Other	105 (47.1%)	17 (16.3%)	33 (68.8%)	55 (77.5%)
Top three major diagnostic categories	Neonates with Genetic Conditions: 97 (43.5%) Nervous System Disorders: 23 (10.3%) Digestive System Disorders: 12 (5.4%)	Neonates with Genetic Conditions: 94 (90.4%) Digestive System Disorders: 1 (1.0%) Hepatobiliary System Disorders: 1 (1.0%)	Nervous System Disorders: 9 (18.8%) Circulatory System Disorders: 6 (12.5%) Metabolic Disorders: 6 (12.5%)	Nervous System Disorders: 13 (18.3%) Mental Disorders: 9 (12.7%) Digestive System Disorders: 7 (9.9%)

NICU: Neonatal intensive care units; PICU: Pediatric intensive care units; NIntCU: Neonatal Intermediate care units

### Clinical outcomes

Patient outcomes during their index hospitalization are presented in Table 2. Overall, average length of stay during the index hospitalization was 42 days (SD = 56) and, on average, results of the CGT was received within 40 days (SD = 32). Children aged between (0–30 days) had a longer length of stay (53 vs. 37 vs. 31; *p* = 0. 032) and a higher inhospital death occurrence (7.7% vs. 0.0% vs. 1.4%; *p* = 0.032) than other age groups. Average time before receiving test results were similar in all groups (40 vs. 43 vs. 37; *p* = 0.674). Regarding the timing of the reception of the CGT results, we found that 69.5% (*n* = 155) were discharged (alive or dead) prior to receiving their results. Among the patients who died during their hospitalization, 67% (*n* = 6) of them received their CGT results before they passed away.

**Table 2:** Patients’ clinical outcomes

Variable	Overall *n* = 223	0–30 days *n* = 104	31 days – 365 days *n* = 48	Above 1 year old *n* = 71	*P*-value
In-hospital death, n (%)	9 (4.0%)	8 (7.7%)	0 (0.0%)	1 (1.4%)	0.032
Length of stay in days, mean (SD)	42 (56)	53 (70)	37 (42)	31 (34)	0.032
More than one genetic test performed, n (%)	62 (27.8%)	24 (23.1%)	8 (16.7%)	30 (42.3%)	0.003
Time before test results in days, mean (SD)	40 (32)	40 (30)	43 (25)	37 (39)	0.674

### Cost analysis

Table 3 shows the average cost for index hospitalization. Results show that patients’ index hospitalizations cost was, on average, $170,337 (95% CI. 128,231 − 219,277). When breaking down these costs into the most important subcategories, we note that, on average, the three most important cost category were related to Clinical care ($118,748 [95% CI. 90,799 − 151,651]; the Hospital’s pharmacy department ($16,418 [95% CI. 9,731 − 26,381]); and the Lab test and medical exams ($8,963 [95% CI. 6,325 − 12,379]).

When comparing patients’ average costs based on key characteristics, we note that the average cost for male inpatients, $192,291 (95% CI. 132,671 − 263,494), was higher than the average cost for female inpatients, but that the difference was not statistically significant (*p* = 0.301) (Table 4). A similar relation was observed when examining costs in function of patients’ age with newborns (0–30 days) costing the most, on average ($219,498 [95% CI. 144,061–312,395]) without identifying any statistically significant increase (*p* = 0.099). In addition, the trends we observed regarding the average cost is similar to the one observed for average cost per day. However, our results did show that the number of CGT conducted within patients’ hospitalization was positively associated total cost of the index hospitalization (average cost of patients who had more than one CGT was $292,305 [95% CI. 171,658 − 441,870] while those who had a single CGT was $123,368 [95% CI. 92,319 − 159,544], p-value = 0.0001).

**Table 3:** Average cost of the index hospitalization

Variable	*n*	Mean (95% CI)	*P*-value
Clinical care			
0–30 days	104	$160,685 (109,813 − 222,248)	0.026
31 days – 365 days	48	$103,882 (58,655 − 157,266)	
Above 1 year old	71	$67,371 (42,968 − 97,281)	
Hospital’s pharmacy			
0–30 days	104	$12,575 (6,672 − 19,764)	0.519
31 days – 365 days	48	$26,161 (5,136 − 64,279)	
Above 1 year old	71	$15,460 (8,407 − 24,803)	
Lab test and medical exams			
0–30 days	104	$9,922 (5,910 − 15,704)	0.874
31 days – 365 days	48	$8,104 (3,061 − 16,221)	
Above 1 year old	71	$8,139 (5,006–12,152)	
Total	223	$170,337 (128,231 − 219,277)	-

**Table 4:** Cost of the index hospitalization stratified by key characteristics

Variable	*n*	Mean (95% CI)	*P*-value
Sex			
Male	119	$192,291 (132,671 − 263,494)	
Female	104	$145,216 (90,989 − 218,038)	0.301
Age^1^			
0–30 days	104	$219,498 (144,061–312,395)	
31 days – 365 days	48	$159,737 (84,531 − 253,590)	
Above 1 year old	71	$105,492 (68,050–152,780)	0.0985
Number of Test CGT			
Patient (n test CGT > 1)	62	$292,305 (171,658 − 441,870	
Patient (n test CGT = 1)	161	$123,368 (92,319 − 159,544)	0.0001
Patients state			
Patients who died during the index hospitalization	9	$352,922 (70,868 − 774,310)	
Patients discharged alive	214	$162,658 (120,889 − 210,894)	0.1451

^1^Average costs per day for the different age-groups are provided in Appendix A

## Discussion

We conducted a cost analysis focussing on a cohort of 223 patients who received CGT after being admitted in CHU Sainte-Justine hospital. These patients were being investigated for a suspected RGD during their inpatient stay. Unstratified results indicate that, on average, their index hospitalization lasted 42 days (SD = 56) and cost $170,337 (95% CI. 128,231 − 219,277). Subgroup analyses revealed that newborns had higher hospitalizations costs, slightly lengthier stays and more inpatient deaths than all other groups.

The average time before obtaining the results of CGT was estimated at 40 days (SD = 32), which aligns with lengths reported by other groups and recommendations of the Canadian College of Medical Geneticists (CCMG) [5]. Even though this time around time respected recommendations by professional organizations, it is likely too long for critically ill patients admitted in all units of hospital and could partially explain their higher hospitalization costs. Furthermore, average cost of patients who had more than one CGT was higher than those who had a single CGT. Difficulties related to patients’ diagnostic and to the degradation of their health state over time could partially explain such observations, but additional work is needed to assess the exact reasons behind such results.

To the best of our knowledge, there exists no other Canadian study that determined the total cost of investigating RGD during a hospitalization stay. The closest Canadian study we are aware of concerns a micro-costing study of genomic testing strategies in autism spectrum disorder, which did not include all resources used during the patients’ hospitalizations [16]. As such, our study fills an important gap in the literature by providing an in-depth analysis of the exact cost of RGD.

More broadly, our results showed that the average cost of the index hospitalization of patients included in our cohort ($170,337 [95% CI: 128,231 − 219,277]) was more than 30 times the average cost of all cause hospitalization of children in Canada (i.e., $4200) [21, 22] and more than 4 time to the results of recent Canadian study [23]. This difference between the results can be explain by the difference of cost parameters and database used in those studies. However our result aligns with past work past Canadian and German that highlighted that patients with suspected rare diseases incurred substantially more costs than their controls [24, 25].

In addition, our results also showed that the costs of RGD differed substantially by age. Indeed, newborns included in our study had statistically significantly higher costs than all other groups. This cost disparity can be partly explained by the fact that a newborn admitted for acute care for a suspected RGD requires more extensive medical interventions, longer hospital stays, and higher resource utilization [24]. Similarly, Glaubitz et al. also demonstrated that the highest costs occurred for infants (< 1 year) and average inpatient costs decreased with age [25].

It is important to note that our study only focused on patients investigated with the use of CGT as ES/GS has only been deployed recently in Quebec. That being said, various studies have hinted that ES/GS could result in substantial cost savings from avoidance of planned tests and procedures which could also reduce patients’ inpatient lengths of stay [26, 27]. For example, Stark et al. report that, although performing WES after exhaustive standard investigation could incur additional costs per diagnosis AU$8,112 (CAN2025 $7,244) [28], if WES was used to replace most of the investigations conducted, it could actually result in saving up to AU$2,182 (CAN2025 $1,948) [28] per additional diagnosis [29]. As such, future work examining the cost of RGD patients investigated with NGS technologies are needed to confirm or refute these claims about their cost saving potential within Canada. This study can therefore serve as a historical baseline of costs to investigate inpatients for RGD prior to the implementation of ES/GS.

### Limitations

It is important to acknowledge limitations in this research. First, data comes from a single site, which could limit the generalization of our results and hinders our potential to assess patients’ follow-up beyond their index hospitalization. On the other hand, the CHU Sainte-Justine is the largest pediatric tertiary care center in Quebec and the second largest in Canada. For this reason, we consider this data to be representative of large pediatric tertiary care centers in Canada and in other publicly funded settings. Second, the CPSS database from which we obtained cost data has been scarcely used in a research context, as it is a relatively new database [30]. Though most cost studies in Canada have used resource intensity weights (RIW) or the NIRRU (identified as Niveau dintensite relative des ressources utilisées) in Quebec, this database was used in this study because the CPSS, unlike these other metrics, provides the actual costs incurred during the patients’ hospitalization from the hospital’s perspective [30–32]. Although it is possible that a micro-costing approach could have provided an even more precise estimate [33], use of the CPSS database to estimate inpatient costs was deemed more feasible and less time consuming while remaining more precise than a general alternative.

## Conclusion

Results of our cost analyses show that pediatric patients included in our CGT cohort incurred costs during their index hospitalization that were over 30 times greater than the average Canadian pediatric hospitalized patient and that cost of newborn patients was even greater. Costs were also higher for patients who had more than one CGT during their stay. Future work will be necessary to evaluate the cost of novel technologies (ES/GS) and their cost-effectiveness profile to guide decision-making on the optimal screening strategy for similar cases. This work can serve as a basis for comparison for such future work.

## Appendix A: Average costs per day for the different age-groups

**Table:** 

Overall	0-30 days	31 days – 365 days	Above 1 year old	*P*-value
$3,039 (2,796 - 3,314)	$3,123 (2,893 - 3,367)	$2,958 (2,301 - 3,852)	$2,971 (2,531 - 3,513)	0.8512

## Data Availability

Deidentified individual data was used in this project. Confidentiality laws in Quebec limit data sharing possibilities.
